# General practitioners' awareness of their own drug prescribing profiles after postal feedback and outreach visits

**DOI:** 10.3109/03009734.2012.713038

**Published:** 2012-10-30

**Authors:** Keld Vægter, Rolf Wahlström, Kurt Svärdsudd

**Affiliations:** ^1^Uppsala University, Department of Public Health and Caring Sciences, Family Medicine and Preventive Medicine Section, Uppsala, Sweden; ^2^Centre for Research & Development in County Sörmland, Sweden; ^3^Karolinska Institute, Division of Global Health (IHCAR), Department of Public Health Sciences, Stockholm, Sweden

**Keywords:** General practice, mailed feedback, outreach visits, prescribing profile

## Abstract

**Background.:**

General practice accounts for the vast majority of drug prescribing in the Nordic countries. Various methods have been used to promote rational drug prescribing. Awareness of own prescribing profile may be a first crucial step in the quality assessment and improvement process.

**Aim of the study.:**

To analyse awareness among general practitioners of their drug prescribing profile during two outreach visits one year apart.

**Methods.:**

All 94 practices with a total of 166 general practitioners in the former Storstrøm County, Denmark, were invited to participate in a project launching outreach visits led by a general practitioner; 88 practices with 160 general practitioners agreed to participate.

**Results.:**

During the first round of outreach visits the general practitioners were asked to rate their own prescribing level of 13 major drug groups as being in the lowest 25%, the middle 26%–74%, or the highest 25% of the distribution across all 88 practices. The result was better than chance (chi-square = 337, 4 df, *r* = 0.37, both *P* < 0.0001). After the assessment a one-hour discussion on rational drug prescribing was held. One year later a new round of outreach visits was held. This time the assessment accuracy was generally greatly improved (chi-square = 724, 4 df, *r* = 0.48, both *P* < 0.0001). The main determinants for the improved accuracy during the second round were high accuracy during the first round, and the number of general practitioners in the practice.

**Conclusions.:**

General practitioners' awareness of their prescribing volumes was substantially improved by a single outreach visit with discussion on rational drug prescribing.

## Introduction

General practice accounts for the vast majority of all drug prescribing in Denmark, as in the other Nordic countries (1,2). Rising reimbursed drug costs have been a major issue in the public debate for decades. Wide variations in the drugs of choice and the volume of drugs prescribed per 1,000 patients have been demonstrated (3). Various methods have been used in order to promote rational drug prescribing among general practitioners (GPs). Little is known about GPs' awareness of their own prescribing profiles, even though such awareness may be a crucial step in the quality assessment and improvement process.

In the early 1990s the local GP community in the former Storstrøm County, Denmark, launched an initiative to encourage GPs to review their prescribing habits in order to improve and enhance rational drug therapy. From 1992 to 1998 prescribing profiles of 13 major drug groups, defined according to the anatomical therapeutic classification system (ATC) (4), were mailed to all 94 practices in the county. In the postal feedbacks, the position of the prescribing volume of each drug group in each practice was given as a percentile of the distribution of prescribing volume of these drug groups across all practices. The information was updated and mailed every 6 months. No other intervention was undertaken.

In a previous publication the effects of the postal feedback were analysed (3). No effects on prescribing habits were found in terms of changed prescribing volume of the drug groups investigated. Possible reasons might have been that the GPs did not read the feedback information, or that they read it but did not pay attention, or that they read it, noticed their position among all practices, but took no action.

In order to check whether the GPs were aware of their prescribing habits, and, if not, to improve their awareness, a second step in the GP initiative, a series of outreach visits, was launched. In this report the effects of these outreach visits on GPs' awareness of their prescribing levels are presented.

## Study population and methods

### Setting

At the time of the study, the former Storstrøm County (since 2007 part of Region Sjælland), was served by 166 general practitioners, distributed across 94 practices. In Denmark general practitioners are private contractors within the national health insurance system, each taking care of approximately 1,500 listed patients. Traditionally most practices in primary health care in Denmark have been solo practices (run by one GP), but over the last decades the formation of group practices has become increasingly common. Each practice is given a specific practice identification number (PIN). All relevant information related to administration and fees in the practices, such as patient demographics, prescriptions, referrals, and specific services and treatments performed in the practice, is registered by the PIN in the County Health Insurance Unit of Statistics for Primary Health Care.

### Data collection

The registration of purchased prescriptions in Denmark offers unique possibilities of following prescribing habits among GPs over time. The ATC system, developed by the World Health Organization (WHO), has been fully implemented in Denmark for decades. All prescriptions filled at Danish pharmacies are registered electronically, and the information stored in the National Danish Medicines Agency database. Information regarding reimbursed drugs was conveyed to the County Health Insurance Unit of Statistics for Primary Health Care and was almost 100% complete.

In 1998, all the 94 practices participating in the postal feedback study in 1992–1998 were invited to participate in an outreach visits programme, involving a 1-hour visit from a GP (K.V., programme facilitator, linked to the programme), of which 88 practices agreed to participate. The outreach visits were performed in two rounds, the first in 1998 and the second in 1999, and followed a predetermined general protocol. First, the programme facilitator gave a brief introduction of the programme and presented a pools coupon-like form, showing the 13 major ATC groups used in the postal feedback. These were antacids (ATC code A02), anti-diabetes drugs (A10), cardiac drugs (C01), diuretics (C03), beta-blockers (C07), calcium channel blockers (C08), reproduction hormones (G03), antibacterial drugs for systemic use (J01), non-steroid anti-inflammatory drugs (M01), analgesics (N02), neuroleptic drugs (N05), psycho-analeptic drugs (N06), and anti-asthma drugs (R03).

The GPs were asked to fill in the form regarding their perception of actual prescribing levels in the practice during the preceding year for each of the 13 ATC groups. Possible responses were the lowest quartile, the top quartile, or the two middle quartiles of the prescribing distribution across all practices. Solo practices gave individual responses, while group practices gave a joint response.

The estimates on the form were then compared with the actual prescribing levels based on register data, and the number of accurate answers registered. During this process rational drug prescribing regarding the drug group in question was discussed in general terms as an important element of the outreach visit. Certain general rules of rational prescribing were stated, such as using generic drug brands when possible, avoiding overuse or underuse of medications, and being generally restrictive about antibiotic prescriptions, and especially regarding the amounts of broad-spectrum antibiotics.

The GP's age and gender, seniority as a GP, number of GPs per practice, access to electronic patient record system, and duration of the outreach visit were recorded. At the end of the session, the GPs filled in an evaluation form regarding the visit, which included their rating of the outreach visit concept in general, rating of the present outreach visit, rating of outreach visits as a quality tool, and their expectations of an outreach visit in a year. In addition, the GP facilitator rated the participating GPs' attitudes to the outreach visit concept and their involvement in the present visit.

In the second round of outreach visits 87 of the 88 practices participated. Updated prescribing data were used, but otherwise the procedure was the same. Since no identifiable GP or patient data were handled there was no need for ethical approval.

### Statistical considerations

Statistical analyses were conducted using the SAS software, release 9.2 (5). Data were complete. Summary statistics, such as means and measures of dispersion, were computed with standard parametric methods.

The assessment of the accuracy of the GPs' estimates of their prescribing profiles poses an analytical problem, since the a-priori probability of an accurate estimate varies with the prescribing level. Those who had an actual prescribing level in the two central quartiles had twice as high a probability of an accurate assessment (50% chance) as those in the lowest and highest quartiles (25% chance each). To overcome this problem the form data, where the responses were given as lowest, two middle, or highest quartile, were cross-tabulated with the actual prescribing levels across all 88 practices graded in the same way. The resulting three-by-three tables were then analysed with the chi-square test, which provided the actual proportion of accurate assessments, the proportion that would be provided by chance only, and the probability that the actual proportions would be better or worse than those provided by chance alone. The same procedure was used for the first and second rounds of outreach visits.

Multivariate linear regression analysis was used to analyse the influence of potential determinants on the change of assessments from the first to the second rounds, using the second assessment as the dependent variable and, as independent variables, the assessment during the first round, GP's age and sex, practice type (solo or group practice), number of years of experience as a GP, number of GPs in the practice, access to electronic patient record system, duration of the outreach visit, the GPs' ratings of the outreach visits, and the GP facilitator's ratings of the participating GPs' attitudes. The multivariate analysis was performed with backward elimination of non-significant variables to avoid model overload. All tests were two-tailed. The level of significance was set at *P* < 0.05.

## Results

### Characteristics of the study population

A total of 48 practices were solo, and 40 were group practices, with an average of 2.8 GPs per practice. The 160 GPs were on average 49.9 years old (interquartile range 46–54 years), 81% were men, and mean GP experience was 15.1 years (interquartile range 11–19 years). There were no significant differences in the distribution of age, sex, and GP experience between GPs in solo versus group practices.

### Results of first and second round of outreach visits

The results of the assessments during the first round of outreach visits are shown in Table I, left hand panel. Regarding antacids (A02) 45 out of 88 (51.1%) practices made an accurate estimate as compared to the 37 (42.0%) expected to provide an accurate estimate by chance only, yielding a significant difference between accurate estimate and estimate by chance (*P* < 0.01). The difference between actual estimate and chance were significant for all drug groups, except anti-diabetes drugs. Across all drug groups, the difference between actual estimate and chance was highly significant (chi-square = 337, 4 df, *r* = 0.37, both *P* < 0.0001). Practices in the lowest and highest quartiles generally made accurate assessments more often (47% accurate versus 24% expected by chance) than those in the middle two quartiles (58% accurate versus 52% expected by chance).

**Table I.** Accurate estimates of general practitioners' own prescribing level in relation to that of all practices in Storstrøm County, Denmark, during the first and second outreach visit round.

		First outreach visit round						Second outreach visit round					
		Observed		Expected				Observed		Expected			
Drug group	ATC code	*n* ^a^	%^b^	*n* ^c^	%^d^	χ^2^	*P* ^e^	*n* ^a^	%^b^	*n* ^c^	%^d^	χ^2^	*P* ^e^
Antacids	A02	45/88	51.1	37/88	42.0	14	<0.01	59/87	67.8	43/87	49.4	32	<0.0001
Anti-diabetes drugs	A10	41/88	46.6	37/88	42.0	5	0.27	58/87	66.7	40/87	46.0	30	<0.0001
Cardiac disease drugs	C01	51/88	58.0	35/88	39.8	21	<0.0005	54/87	62.1	41/87	47.1	22	<0.0005
Diuretics	C03	43/88	48.9	33/88	37.5	18	<0.005	61/87	70.1	40/87	46.0	43	<0.0001
Beta-blockers	C07	38/88	43.2	31/88	35.2	12	<0.05	46/87	52.9	35/87	40.2	12	<0.05
Calcium channel blockers	C08	48/88	54.6	36/88	40.9	15	<0.005	52/87	59.8	36/87	41.4	20	<0.001
Reproduction hormones	G03	51/88	58.0	33/88	37.5	27	<0.0001	62/87	71.3	37/87	42.5	51	<0.0001
Antibiotics	J01	46/88	52.3	32/88	36.4	24	<0.0001	58/87	66.7	34/87	39.1	45	<0.0001
NSAIDs ^f^	M01	54/88	61.4	34/88	38.6	39	<0.0001	59/87	67.8	36/87	41.4	44	<0.0001
Analgesics	N02	52/88	59.1	34/88	38.6	29	<0.0001	54/87	62.1	40/87	46.0	20	<0.001
Neuroleptics	N05	41/88	46.6	30/88	34.1	13	<0.01	51/87	58.6	29/87	33.3	42	<0.0001
Antidepressants	N06	46/88	52.3	34/88	38.6	18	<0.001	65/87	74.7	35/87	40.2	66	<0.0001
Anti-asthma drugs	R03	48/88	54.6	32/88	36.4	17	<0.005	62/87	71.3	40/87	46.0	51	<0.0001
All drug groups		604/1144	52.8	438/1144	32.3	197	<0.0001	741/1131	65.5	485/1131	42.9	417	<0.0001

^a^Number of accurate estimates of all estimates made.

^b^Proportion of accurate estimates.

^c^Number of accurate estimates expected by chance only of all estimates made.

^d^Proportion of accurate estimates expected by chance only.

^e^ *P* for difference observed–expected.

^f^Non-steroid anti-inflammatory drugs.

The corresponding data for the second round are shown in the right hand panel of Table I. The assessments generally showed a higher degree of accuracy in relation to what could be expected by chance than those from the first round. The assessment accuracy improved especially for anti-diabetes drugs, now highly significant. Not only the assessments in the extreme quartile practices improved (60% accurate versus 22% expected by chance), but also those in the combined middle two quartiles (69% accurate versus 56% expected by chance). Overall, the estimations during round two were better than those during round one (chi-square = 724, 4 df, *r* = 0.48, both *P* < 0.0001).

### Change from the first to the second outreach visit

**Figure 1.**
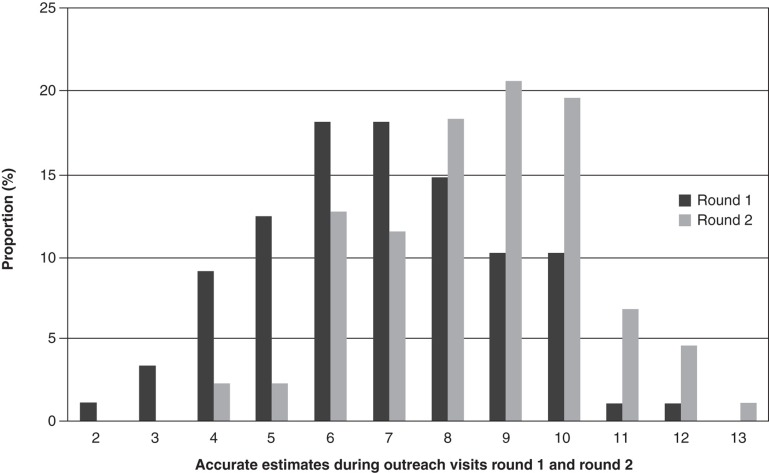
Distribution of number of accurate estimates of practices' prescribing position (lowest quartile, the top quartile, or the two middle quartiles in the prescribing level distribution across all practices and across all drug groups) during the first and second round of outreach visits.

**Figure 2.**
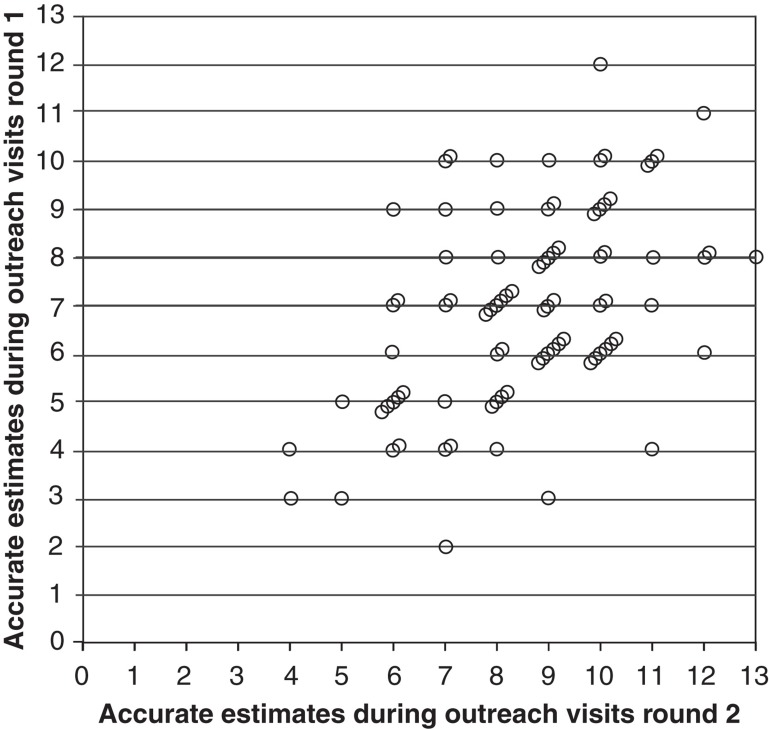
Change of accurate estimates of individual practices' prescribing position (lowest quartile, the top quartile, or the two middle quartiles in the prescribing level distribution across all practices and across all drug groups) from the first to the second round of outreach visits.

The improvement of accurate assessments from the first to the second outreach visits across all participating practices and across all drug groups is shown in the bar graph in Figure 1. There was a clear shift in the distribution of number of accurate assessments from round one to round two. The scatter plot in Figure 2 shows the results based on individual practices. The number of accurate assessments during round one is shown on the vertical axis and those from round two on the horizontal. The diagonal line indicates no change. A shift of dots from the upper left half of the graph to the lower right one indicates a movement towards more accurate assessments.

To find determinants of the difference in accurate assessments between the two rounds a multivariate linear regression was performed, with the proportion of accurate assessments in the second round as the dependent variable and, as the independent variables, the proportion of accurate estimates from the first round, GP's age and sex, practice type (solo or group practice), number of GPs in the practice, number of years of experience as a GP, access to electronic patient record system, duration of the outreach visits, GPs' ratings of the outreach visits, and the GP facilitator's ratings of the participating GPs' attitudes. The only significant determinants affecting the degree of accurate assessments during round two were the degree of accurate assessments during round one (regression coefficient = 0.41, *F* = 22.72, *P* < 0.0001), and the number of GPs in the practice (regression coefficient = 0.42, *F* = 7.35, *P* < 0.01), both increasing the degree of accuracy in the second round.

## Discussion

GPs' assessments of their prescribing profiles were generally better than chance during the first round of outreach visits, and were even better during the second. The majority of the practices improved their assessment accuracy, while a minority had no change or had fewer accurate assessments during round two. The two most important determinants of an improvement from the first to the second round were good results during the first round and the number of GPs in the practice.

The strengths of the study include that the actual prescribing levels were based on register data with a high degree of completeness and reliability, and that the response form might be regarded as having high face validity. All practices within a geographically defined area were invited to participate; the non-participation rate, 6 out of 94 practices (6.4%), was low and is unlikely to have affected the results. A limitation might be that the data referred to prescribing habits during the 1990s and not today. However, the problems regarding rational prescribing habits are still prevalent (6). We therefore have no reason to believe that the results are biased to such an extent that the conclusions would be affected.

In a previous report from this study it was found that postal feedback of prescribing profiles regarding the same 13 drug groups as used in this study had no effect on prescribing volumes, in spite of the fact that the project was initiated by the local GP community and that the information was updated and sent out every 6 months for 7 years (3). We speculated on possible reasons for the lack of effect on prescribing habits, for instance that the feedback information was not read, or was read but ignored, or was read but no action taken.

The results from the present study indicate that the first option, that the feedback was not read, probably does not apply. Since the GP assessments during the first round were generally more accurate than chance, they most probably read the feedback information. However, this information did not constitute a sufficiently strong argument for the GPs to take action. More of the same would probably not have made any difference, since a total of 14 semi-annual feedback sheets had no effect on prescribing habits.

Other means appear to be necessary to achieve changes in prescribing habits. In this study, outreach visits were used. There was a clear effect of the visits during the second round. The improved results were most probably caused by the discussions during the first round, since neither we nor anyone else made any other intervention. The content of these discussions focused on the prime purpose of the project, to promote rational drug prescribing.

The two rounds of outreach visits in this study thus appear to have had an effect on the GPs' awareness of their prescribing habits. Whether it also affected the prescribing habits is not known. However, outreach visits have been shown to affect prescribing habits in other studies. In a Canadian trial, 54 GPs whose prescribing of analgesics was more than two standard deviations above average were randomly allocated to receiving notification of their prescribing volume and a 1-day group education activity, or to receiving written notification only, or to no intervention (6). Those in the first group decreased their prescribing volume by 33%, and those in the second group by 25%, while there was no change in the third group. Similar but smaller effects were found in three Norwegian studies (7-9) and in another Canadian study (10), when written feedback of prescribing profiles was combined with treatment recommendations. A 2006 Cochrane review stated that the combination of audit and feedback only had a small to moderate effect on professional practice (11).

In this study the outreach visit with discussion of general guidelines for rational drug prescribing may be regarded from the individual practice point of view as an isolated educational opportunity. More long-term educational efforts are probably needed to achieve larger, more sustainable effects.

In conclusion, after 7 years of semi-annual feedback information on prescribing level of 13 drug groups, GPs rated their levels of prescribing in relation to the prescribing level distribution across all practices better than chance. After a single educational session during an outreach visit, their knowledge of their own prescribing levels was further improved.
